# Comparison of peripheral refraction and higher-order aberrations between orthokeratology and multifocal soft contact lens designed with highly addition

**DOI:** 10.1007/s00417-022-05573-1

**Published:** 2022-02-22

**Authors:** Yingying Huang, Xue Li, Chenglu Ding, Yunyun Chen, Xinjie Mao, Hao Chen, Jinhua Bao

**Affiliations:** 1 Eye Hospital and School of Ophthalmology and Optometry, Wenzhou Medical University, 270 West Xueyuan Road, Wenzhou, 325027 Zhejiang China; 2 National Clinical Research Center for Ocular Diseases, Wenzhou, Zhejiang China

**Keywords:** Multifocal soft contact lens, Orthokeratology, Peripheral myopic defocus, Higher-order aberrations, Contrast visual acuity

## Abstract

**Purpose:**

To compare peripheral defocus, higher-order aberrations (HOAs), and contrast visual acuity (CVA) in myopic children wearing orthokeratology (OK) lenses and multifocal soft contact lenses (MSCLs) designed with highly addition.

**Methods:**

This is a prospective, nonrandomized, controlled study. Subjects at 8 to 13 years of age with spherical equivalent refraction from − 1.00 to − 5.00 dioptres (D) were included in the OK group (*n* = 30) and MSCL group (*n* = 23). Relative peripheral corneal defocus (RPCD) and relative peripheral refraction (RPR) were measured before and after wearing lenses. HOAs including spherical aberration (SA), coma, trefoil, and total HOAs, and high (100%) and low (10%) CVA were compared between the groups. Axial length (AL) was measured before and after wearing the lenses for 1 year.

**Results:**

After wearing the lenses, subjects in the MSCL group had RPCD and RPR values similar to the OK group at the paracentral (within 2 mm of the cornea or 20° of the retina, all *p* > 0.05) but larger than the OK group at the periphery (all *p* < 0.05). All HOAs increased after wearing the lenses except the trefoil in the MSCL group (all *p* < 0.05). HOAs increased more in the OK group (all *p* < 0.05). The 100% and 10% CVAs were worse in the MSCL group (*p* = 0.02 and *p* = 0.004). After 1 year, AL elongation was 0.37 mm (SD = 0.16) in the MSCL group and 0.28 mm (0.16) in the OK group (*p* = 0.06).

**Conclusion:**

MSCL produced larger myopic defocus at the periphery, increased less HOAs and had worse CVA than OK lens. The high addition of this MSCL did not result in better myopia control efficacy

**Trial registration:**

Chinese Clinical Trial Registry: ChiCTR1800018564. Registered 25 September 2018; retrospectively registered, http://www.chictr.org.cn/showproj.aspx?proj=31376


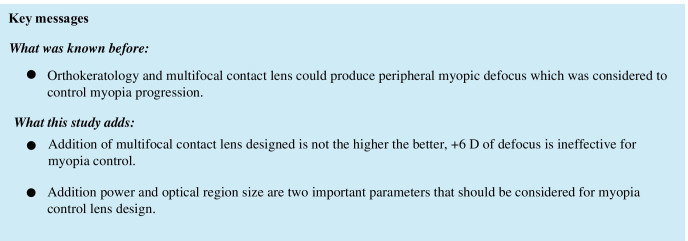


## Introduction

Myopia has become a global pandemic in recent decades [1]. Myopia can develop quickly during primary school ages, and some individuals will develop high myopia in adulthood. The incidence of high myopia is increasing year by year, and some cases are sufficiently serious to cause blindness due to pathological myopia, such as retinal detachment, glaucoma, and myopic choroidal neovascularization [2, 3]. Thus, controlling myopia in children of primary school age is important and necessary.

Orthokeratology (OK) lenses are rigid gas permeable contact lenses with a reverse-geometry design and are intended to be worn at night. OK lens is a common clinical myopia control approach [4]. A meta-analysis showed OK lenses could slow myopia progression by approximately 30% to 60% [4]. Another efficient method that is widely recognized is bifocal (BSCL) or multifocal soft contact lenses (MSCL) [5–7]. These are commonly designed for central distance correction and peripheral additions.

Previous animal studies found that relative peripheral hyperopia induced by a negative lens produces central axial elongation, whereas peripheral myopic defocus produces axial hyperopia [8–11]. This method has been applied in children for myopia control, as peripheral defocus is proposed to be one of the mechanisms by which OK lenses, BSCLs, and MSCLs slow myopia progression [12, 13]. Previous studies of children with low to moderate myopia measured horizontal and vertical peripheral defocus after wearing OK lenses. They found that the lenses turned peripheral hyperopic defocus to myopic defocus and sustained it during the wearing period [14, 15]. BSCLs and MSCLs also produce different magnitudes of peripheral myopic defocus with different additions [16–18]. Peripheral defocus and other optical changes caused by post-OK or wearing MSCL also cause changes in the corneal and retinal higher-order aberrations (HOAs). Several previous studies found a significant increase in spherical aberration (SA), coma, and total HOAs [17, 19, 20]. An increase in HOAs also causes a decrease in visual quality, noted as a decrease in contrast sensitivity or contrast visual acuity (CVA), which may influence the daily life of children [21–23].

Previous studies found that BSCLs and MSCLs significantly slowed axial elongation. However, different optic designs and additions lead to different myopia control efficacies [5]. In previous studies, BSCLs and MSCLs were commonly designed with low to moderate additions (+ 0.50 to + 4.00 diopter [D]), which produced relatively lower retinal peripheral myopia defocus than that from OK lenses [5, 6, 13, 18, 24, 25]. However, animal studies suggested that a higher peripheral myopic defocus had a better ability to maintain hyperopia, slow the myopia progression, or counteract the myopiagenic effect [26, 27]. MSCLs of the same design with different levels of add powers also showed that higher addition had a better effect on myopia control in children [28]. Thus, it was hypothesized that lower addition limits the myopia control effect of BSCLs and MSCLs.

A new MSCL designed to mimic the optical performance established in OK lens with highly addition has been recently introduced for myopic control in the clinic [29]. We performed this prospective, nonrandomized, control study to compare peripheral defocus, aberrations, and contrast visual acuity in children wearing MSCL with OK lens and to show the 1-year myopia progression.

## Methods

This study was approved by the institutional review board of the Eye Hospital of Wenzhou Medical University, and all work was carried out in accordance with the tenets of the declaration of Helsinki. Consent was obtained from the children and their guardians after verbal and written explanations of the objectives and possible consequences of the study. The inclusion criteria were age between 8 and 13 years old, spherical equivalent refraction (SER) between -1.00 D and -5.00 D, less than 0.75 D of astigmatism, less than 1.00 D of anisometropia, in good ocular health, and free from systemic disease. At the Eye Hospital of Wenzhou Medical University, eligible subjects were recruited into the MSCL group (*n* = 23) or the OK group (*n* = 30) based on the personal desires of the children and their guardians. All measurements were obtained from the right eye.

The MSCL (Softok, ArtMost, Taiwan, China) was specifically designed to mimic the optical design of OK and produce a large amount of peripheral myopic defocus [29]. The lens material was ocufilcon D, the total lens diameter was 14.4 mm, and the base arc radius was 9 mm. The central optical zone for distance correction was 6 mm, and the peripheral optical zone was designed with high addition [30]. The OK lenses (Euclid System Corp., Herndon, VA, USA) were worn overnight (Boston Equalens II), and the total lens diameter was 10.6 mm with a 6.2-mm optic zone diameter.

Corneal topographies were performed with a Scheimpflug corneal topographer (Sirius, CSO, Florence, Italy, csoitalia.it). Refraction of the anterior corneal surface was obtained from the sagittal anterior refractive power map, and relative peripheral corneal defocus (RPCD) was defined as the peripheral refraction minus the apex corneal refraction. RPCD was obtained at 1 mm, 2 mm, 3 mm, and 4 mm at nasal (N), temporal (T), superior (S), and inferior (I) cornea; S4 (4 mm at superior cornea) was not available from some subjects because of upper eyelid occlusion and was excluded from the analysis.

Wavefront aberration measurements were performed using a Shack-Hartmann aberrometer (WASCA Analyser, Zeiss, Saalfeld, German, zeiss.com) through the nondilated pupil in the dark. Analyses were performed on 5 mm pupils, and Zernike coefficients up to the 7th order were reported using the Optical Society of America standards. The Zernike coefficients were used to calculate the root mean squared (RMS) error for the total HOAs (3rd to 7th order), coma (C_3_^−1^ and C_3_^1^), trefoil (C_3_^−3^ and C_3_^3^), and SA (C_4_^0^).

CVA was evaluated monocularly (right eye) at 5.5 m using a multifunctional visual acuity test (MFVA-100, Shenzhen BriteEye Medical Tech, Shenzhen, China, 986,875.51sole.com) [31] with MSCL or post-OK under photopic conditions (average illumination in front of the right eye = 200 lx). High (100%) and low (10%) CVAs were tested and reported as the log minimum angle of resolution (logMAR).

Peripheral refraction (PR) was obtained from an open-field Grand Seiko binocular autorefractor (WAM-5500, Rexxam Co. Ltd., Kagawa, Japan, grandseiko.com) after cycloplegia (two drops of 1% cyclopentolate). Refractive errors were measured at central 0° (primary gaze) and horizontal peripheral 10°, 20°, and 30° for nasal (N) and temporal (T) retinal eccentricity. Relative peripheral refraction (RPR) was determined by subtracting the central refraction values from the PR values.

Axial length (AL) was measured by Lenstar ocular biometry (LS900, Haag-Streit International, Koeniz, Switzerland, haag-streit.com). Five individual measurements with differences of no more than 0.02 mm were obtained and averaged. Subjects in the OK group were asked to discontinue for 1 month for cornea recovering, so AL was measured at baseline and 12 months after wearing MSCLs or 13 months after wearing OK lenses.

**Fig. 1:**
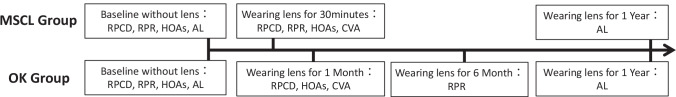
Measurement schedule in the two groups. OK, orthokeratology; MSCL, multifocal soft contact lens; RPCD, relative peripheral corneal defocus; RPR, relative peripheral refractive error; HOAs, higher-order aberrations; CVA, contrast visual acuity; AL, axial length

RPCD, RPR, and HOA measurements were performed at baseline and after wearing the MSCLs for 30 min or after wearing the OK lenses for 1 month (Fig. 1). Since the RPR was stable while wearing an OK lens for 1 to 12 months [32], and the subjects needed to undergo a dilation examination at the 6-month follow-up (the measurements and results are not shown in this paper), the RPR examination was performed at the 6-month follow-up instead of the 1-month follow-up to avoid adding an additional dilation. AL was measured at baseline, 6 months, and 1 year. The data were tested for a normal distribution.

Repeated measures ANOVA was used to compare the differences in before and after wearing lens and different treatments; baseline refractive error was corrected when compared between groups. When appropriate, post hoc *t*-test was used to compare the differences for each parameter of RPCD, RPR, HOA, and CVA. The comparison of AL elongation was adjusted for baseline age, sex, and refractive error. Multiple regression was performed between CVA and HOAs after wearing MSCL or post-OK in two group. Statistical significance was determined at the *p*-values < 0.05 that were adjusted for multiple comparisons.

## Results

Comparison of the baseline characteristics showed that subjects in the OK group were less myopic than those in the MSCL group (Table 1). Age, sex ratio, and axial length showed no significant difference between the groups.

**Table 1:** Baseline characteristics in two groups presented as mean (SD)

Parameters	OK group	MSCL group	*p* value
Age	9.90 (1.27)	9.70 (1.49)	0.55
Gender (M/F)	13/17	10/13	0.99
Refractive error (D)	− 2.63 (0.71)	− 3.18 (0.71)	0.008*
Axial length (mm)	24.89 (0.91)	24.88 (0.72)	0.97

*OK* orthokeratology, *MSCL* multifocal soft contact lens, *p < 0.05.

**Fig. 2:**
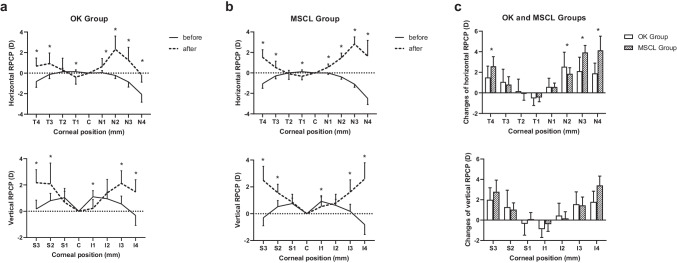
RPCD in the OK (**a**) and MSCL (**b**) groups before and after wearing lenses and comparisons of the changes in the RPCD in the two groups (**c**). Both the horizontal RPCD (top row) and the vertical RPCD (bottom row) were included. Error bars represent 1 SD of the mean. RPCD, relative peripheral corneal defocus; OK, orthokeratology; MSCL, multifocal soft contact lens; D, dioptre; N, nasal cornea; C, central; T, temporal cornea; S, superior cornea; I, inferior cornea; *p < 0.05

RPCD became more positive after wearing OK lenses and MSCLs (Fig. 2, both *p* < 0.001). Horizontal RPCD at all N sides and T3, T4, and vertical RPCD at S2, S3, I3, and I4 became more positive and caused myopic defocus at the retina (all *p* < 0.05). Comparison of the changes of RPCD between the groups showed significant difference in the horizontal (*p* = 0.04) but not in the vertical (*p* = 0.16). N2 had more positive changes after wearing the OK lens than the MSCL (*p* = 0.03), while N3, N4, and T4 had more positive changes in the MSCL group than in the OK group (all *p* < 0.05).

**Fig. 3:**
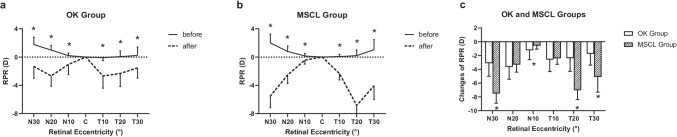
RPR in the OK (**a**) and MSCL (**b**) groups before and after wearing the lenses and a comparison of changes in the two groups (**c**). Error bars represent 1 SD of the mean. RPR, relative peripheral refraction; OK, orthokeratology; MSCL, multifocal soft contact lens; D, dioptre; N, nasal retinal; C, central; T, temporal retinal; *p < 0.05

One subject in the MSCL group did not finish the RPR measurement after wearing lens; the analysis included only 22 subjects in the MSCL group. The horizontal RPR changed from hyperopic defocus to myopic defocus after wearing the OK lens and MSCL (Fig. 3, both *p* < 0.001). The largest defocus was at T10 (− 2.68 [1.71] D) in the OK group and at T20 (− 6.84 [1.47] D) in the MSCL group. Comparison of the changes of RPCD between the groups showed significant difference (*p* < 0.001). RPR became more myopic at N30, T20, and T30 in the MSCL group than in the OK group (all *p* < 0.05).

**Fig. 4:**
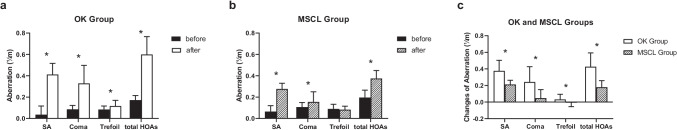
HOAs in the OK (**a**) and MSCL (**b**) groups before and after wearing the lenses and a comparison of changes between the two groups (**c**). Error bars represent 1 SD of the mean. OK, orthokeratology; MSCL, multifocal soft contact lens; SA, spherical aberration; HOAs, higher-order aberrations; *p < 0.05

One subject in the OK group and three subjects in the MSCL group did not complete the wavefront aberration exam, so the HOA analysis included only 29 OK subjects and 20 MSCL subjects. HOAs significantly changed after wearing OK and MSCL lenses (both *p* < 0.001). SA, coma, trefoil, and total HOAs significantly increased after 1 month of wearing the OK lens (all *p* < 0.01, Fig. 4). In the MSCL group, SA, coma, and total HOAs increased with lens wearing (all *p* < 0.05). The changes in the HOAs were significantly different between the two groups (*p* < 0.001). SA, coma, trefoil, and total HOAs all increased more in the OK group than in the MSCL group (all *p* < 0.05).

**Fig. 5:**
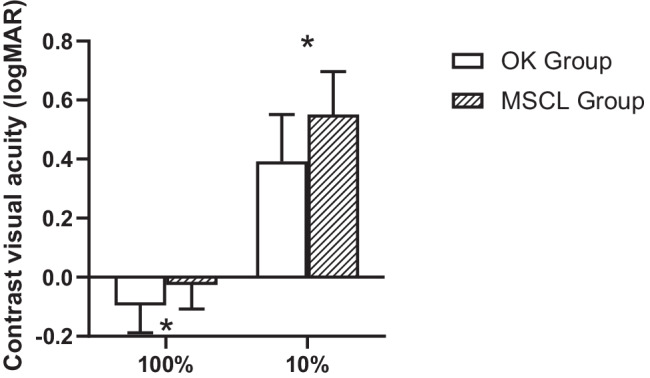
Comparison of high (100%) and low (10%) contrast visual acuity in the two groups after wearing the lenses. Error bars represent 1 SD of the mean. OK, orthokeratology; MSCL, multifocal soft contact lens; *p < 0.05

Subjects with OK lenses had better CVAs than those with MSCL (Fig. 5, *p* < 0.001), i.e., 0.06 logMAR different for 100% contrast (*p* = 0.02) and 0.14 logMAR different for 10% contrast (*p* = 0.004). Total HOA was found to be positively correlated with CVA 10% in the OK group (*r* = 0.48, *p* = 0.01), but no correlation was found between HOA and CVA in the MSCL group.

In the MSCL group, four subjects dropped out during the 1-year follow-up, and 19 subjects were included for the AL analysis. After 1 year, myopia progression was 0.37 (0.16) mm in the MSCL group and 0.28 (0.16) mm in the OK group (*p* = 0.06). After adjusting for baseline sex, age, and refractive error, myopia progression was 0.36 (0.16) mm in the MSCL group and 0.28 (0.15) mm in the OK group (*p* = 0.12).

## Discussion

The largest addition of previous designs of BSCLs and MSCLs only caused -1 to -2 D of peripheral myopic defocus at the retina [33–35]. This amount is lower than the defocus caused by OK lenses (-2 D to -3 D) [35]. The highly addition designed MSCL used in this study was found to have the same defocus at paracentral with the OK lens (within 2 mm of the cornea or 20° of the retina), but a higher defocus at the periphery than the OK lens. The largest myopic defocus in the MSCL group was more than 6 D in the retina (T20). As far as we know, no soft contact lens, OK lens, or spectacles have previously reached this high amount of peripheral defocus. However, the MSCL did not show slower myopia progression than the OK lens in this study or in previous studies [4], and did not show slower myopia progression than other multifocal/bifocal lenses in previous studies [6, 13, 18, 36, 37].

Compared with two long-term effective myopia control MSCLs, the MiSight [7, 36] and defocused incorporated soft contact (DISC) lenses [6], the lenses used in this study had a larger central zone (MiSight, 3.36 mm; DISC, 3 mm; vs. current study, 6 mm) and a higher add power (MiSight, + 2.0 D; DISC, + 2.5 D; vs. current study, + 8 to + 20 D) [6, 28, 30]. Thus, there are two conjectures about lens design to explain the ineffectiveness of the MSCL in this study. One is that highly myopic defocus is beyond the ability of the retina to detect the defocus or the sign of the defocus. In tree shrew eyes, Norton et al. arranged a series of positive lenses to compete against a myopiagenic -5 D lens [11]. They found that + 5 D lenses had the highest effectiveness, while the myopic defocus provided by + 6 D and + 10 D lenses was ineffective in competing against hyperopic defocus [11]. Similarly, there may also be a limited range of myopic defocus that the human retina can detect. The high defocus produced by MSCL in this study may be out of this range. Another conjecture is the large central optical zone. The MSCL used in this study had a 6 mm central optical zone to correct the distance refraction, while the central zones of the previous bifocal or multifocal lenses were usually less than 4 mm [13, 24, 25, 38]. The largest RPCD of the OK lens was found at N2/T3/I3/S3, while the largest RPCD of the MSCL was more at the periphery, i.e., N3/T4/I4/S3. Similarly, the largest RPR was at T10 in the OK group but more at the periphery (T20) in the MSCL group under the dilated pupil. T3/N4/S4/I3 and T20 were larger than the natural pupil size, and light from the addition area may be hard to refract into the retina [9, 39].

A positive shift in SA, coma, and total HOAs was found after wearing the OK lens and MSCL, and the result is consistent with previous studies [17, 19, 20]. However, the MSCL group had worse CVA than the OK group. Berntsen et al. reported that SA was the main contributor to worse CVA in subjects with OK lenses [20]. In this study, coma and total HOAs were correlated with low CVA in the OK group, and no correlation was found in the MSCL group. It was speculated that the dry eye after wearing a soft contact lens influences visual acuity.

One of the limitations of this study is the control nonrandomized design; subjects selected treatments according to the wishes of their own and their caregivers, which resulted in a 0.50 D difference in baseline refractive error between the groups. Correlation analysis between baseline refractive error and AL elongation, RPCD, RPR, and HOAs after wearing lenses did not show any significance except N30 of RPR in the OK group (*r* = 0.47, *p* = 0.009) and T30 of RPR in the MSCL group (*r* = 0.56, *p* = 0.007). The differences of N30 and T30 between groups were large enough that it can be considered that the unequal baseline refraction was not enough to affect the statistical results. Also, either of them had a significant correlation with AL elongation. Thus, baseline difference of refractive error was considered not significantly influence the results. Another limitation is that the CVA was not measured with a single vision lens before wearing the OK lens or MSCL to exclude intersubject differences; the difference in CVA between groups could not be fully attributed to the lenses. Last is that the influence of decentration of MSCL and OK lens was not included in the analysis of outcomes, but subjects in the OK group showed good centration by fluorescein staining evaluation.

## Conclusion

MSCLs designed with highly addition produced the same defocus at the paracentral region but higher defocus at the periphery than OK lenses and a higher addition than any previous multifocal lenses. HOAs increased less in the MSCL group than in the OK group, but the CVA was worse in the MSCL group. Low CVA was positively correlated with HOAs in the OK group but was not correlated with CVA in the MSCL group. The high addition of this MSCL did not result in better myopia control efficacy, and future research needs to explore the best design of the multifocal contact lens.

## Data Availability

The datasets used and analyzed for the present study are available from the corresponding authors upon reasonable request.
